# Network pharmacology and experimental analysis to reveal the mechanism of Dan-Shen-Yin against endothelial to mesenchymal transition in atherosclerosis

**DOI:** 10.3389/fphar.2022.946193

**Published:** 2022-08-24

**Authors:** Mengyun Hong, Yubiao Wu, Haiyi Zhang, Jinchao Gu, Juanjuan Chen, Yancheng Guan, Xiude Qin, Yu Li, Jiahui Cao

**Affiliations:** ^1^ The Research Center of Basic Integrative Medicine, Guangzhou University of Chinese Medicine, Guangzhou, China; ^2^ Encephalopathy Department, Shenzhen Traditional Chinese Medicine Hospital, The Fourth Clinical Medical College of Guangzhou University of Chinese Medicine, Shenzhen, China; ^3^ Obstetrics and Gynecology Department, Shenzhen Traditional Chinese Medicine Hospital, The Fourth Clinical Medical College of Guangzhou University of Chinese Medicine, Shenzhen, China; ^4^ Nursing Department, Guangzhou University of Chinese Medicine, Guangzhou, China

**Keywords:** endothelial to mesenchymal transition, Dan-Shen-Yin, atherosclerosis, PI3K/AKT signaling, LASP1, network pharmacology

## Abstract

Atherosclerosis is a chronic inflammatory disease characterized by the formation of plaque and endothelial dysfunction. Under pro-inflammatory conditions, endothelial cells adopt a mesenchymal phenotype by a process called endothelial-to-mesenchymal transition (EndMT) which plays an important role in the pathogenesis of atherosclerosis. Dan-Shen-Yin (DSY) is a well-known traditional Chinese medicine used in the treatment of cardiovascular disease. However, the molecular mechanism whereby DSY mitigates atherosclerosis remains unknown. Therefore, we employed a network pharmacology-based strategy in this study to determine the therapeutic targets of DSY, and *in vitro* experiments to understand the molecular pharmacology mechanism. The targets of the active ingredients of DSY related to EndMT and atherosclerosis were obtained and used to construct a protein-protein interaction (PPI) network followed by network topology and functional enrichment analysis. Network pharmacology analysis revealed that the PI3K/AKT pathway was the principal signaling pathway of DSY against EndMT in atherosclerosis. Molecular docking simulations indicated strong binding capabilities of DSY’s bioactive ingredients toward PI3K/AKT pathway molecules. Experimentally, DSY could efficiently modify expression of signature EndMT genes and decrease expression of PI3K/AKT pathway signals including integrin αV, integrin β1, PI3K, and AKT1 in TGF-β2-treated HUVECs. LASP1, which is upstream of the PI3K/AKT pathway, had strong binding affinity to the majority of DSY’s bioactive ingredients, was induced by EndMT-promoting stimuli involving IL-1β, TGF-β2, and hypoxia, and was downregulated by DSY. Knock-down of LASP1 attenuated the expression of integrin αV, integrin β1, PI3K, AKT1 and EndMT-related genes induced by TGF-β2, and minimized the effect of DSY. Thus, our study showed that DSY potentially exerted anti-EndMT activity through the LASP1/PI3K/AKT pathway, providing a possible new therapeutic intervention for atherosclerosis.

## Introduction

Atherosclerosis (AS) is a progressive inflammatory disease triggered by numerous systemic risk factors, including smoking, diabetes, hyperlipidemia, hyperglycemia, hypertension and disturbed blood flow in local areas. These stimuli promote hardening and thickening of artery walls as well as the formation of plaques. Upon formation, plaques can result in intravascular thrombosis and interruption of blood flow, potentially leading to morbidity or death. Studies have shown that plaque formation is associated with the accumulation of mesenchymal cells in the arterial intima, which secrete pro-inflammatory molecules and synthesize extracellular matrix and metalloproteinases (Stoll and Bendszus 2006; Flores-Gomez et al., 2021). Although in past decades, the mesenchymal cells in the tunica intima have been widely studied, their origin is not well understood. Under pathological conditions, smooth muscle cells in the tunica media and fibroblasts in the tunica adventitia proliferate and migrate into the tunica intima leading to the thickening of the intima (Xu et al., 2007; Tillie et al., 2020; Miano et al., 2021). In addition, bone marrow-derived cells were also involved in tunica intimal thickening (Cochain and Zernecke 2017). More recently, studies of human, porcine, and mouse arterial plaques have shown that the mesenchymal cells in the tunica intima could be derived from endothelial cells (Chen et al., 2015; Mahmoud et al., 2017; Helmke et al., 2019).

Such cells are considered to arise from the transitional state of the endothelial to mesenchymal transition (EndMT), in which endothelial markers continue to be expressed and new mesenchymal markers are acquired. EndMT was first described during heart development and was characterized morphologically as representing a shift from a cobblestone-like shape to a spindle morphology accompanied by alterations in gene expression profiles (Markwald et al., 1977). EndMT has recently been suggested as a fundamental contributing factor to AS. In an *in vivo* lineage tracing experiment, the endothelial-specific Cre-lox system VE-cadherin-CreERT2 or SCL-CreERT2 in combination with ApoE^−/−^ or LDLR^−/−^ mouse AS models were used to study the deep relationship between EndMT and AS (Chen et al., 2015; Evrard et al., 2017). Approximately 30% of aortic endothelial cells underwent EndMT, as assessed by co-expression of lineage tracers and the mesenchymal markers, Notch3 (Chen et al., 2015) and FAP (Evrard et al., 2017) after 4 months of on a high-fat diet. Meanwhile, 50% of endothelial-derived mesenchymal cells completely lost the expression of VE-cadherin (Evrard et al., 2017), indicating that these cells had undergone a complete EndMT. Other researchers proved that EndMT was associated with the occurrence of AS (Chen et al., 2015; Anbara et al., 2020; Qin et al., 2020), and the degree of EndMT was closely related to the severity of atherosclerotic disease (Chen et al., 2015), suggesting that the EndMT process has important clinical significance. This evidence demonstrates that the EndMT process can serve as a potential target for the treatment of AS.

Dan-Shen-Yin (DSY) comes from the Shi Fang Ge Kuo and is comprised of red sage (*Salvia miltiorrhiza*), sandalwood and fructus amomi (amomi fruit). As a famous traditional Chinese formula, DSY has been considered to improve blood circulation in Chinese theory and has been prescribed for centuries to treat coronary artery disease. Studies have indicated that DSY exerted protective effects against inflammation and reduced oxidative stress in a rat model of diabetic AS (Yan et al., 2011) and acute ischemic myocardial injury (Yan et al., 2012). Furthermore, a modified DSY composed of red sage (*Salvia miltiorrhiza*), sandalwood, Chuanxiong rhizoma, carthami flos, Rehmanniae radix preparata, and *Angelica sinensis* radix showed beneficial therapeutic effects against AS in an ApoE^−/−^ mouse model (Yang et al., 2020). The active ingredients in *Salvia miltiorrhiza*, including tanshinone IIA (Wang et al., 2017), dihydrotanshinone I (Zhao W. et al., 2016) and salvianolic acid A (Ma et al., 2020), could efficiently treat AS. It was reported that sandalwood extract exhibited anti-inflammatory activity and could attenuate hyperlipidemia in streptozotocin-induced diabetic rats (Kulkarni et al., 2012; Suganya et al., 2021). Fructus amomi has also been found to display anti-inflammatory properties (Zhang T. et al., 2017; Fan et al., 2022). Due to the fact that inflammation affects the EndMT process, we hypothesized that DSY could target EndMT to treat AS.

Traditional Chinese medicines have gained much therapeutic interest in treating AS because of their multiple components with multiple targets; but their underlying mechanisms have been difficult to decipher. Based on the rationale of ‘disease-gene-target-medicine’, network pharmacology has been extensively used to understand the complicated mechanisms of traditional Chinese medicine (TCM) in treating complex diseases. In this work, we used network pharmacology and experimental strategies to investigate and elucidate the mechanism of DSY’s effects on EndMT to support the rationale of using DSY in treating AS. We obtained the ingredients of DSY with the help of several databases and screened them for their EndMT and AS targets, which were acquired with multi-source databases and literature. We then set up protein-protein interaction (PPI) networks to determine the interactions among these targets and in turn performed enrichment analysis using gene ontology (GO) and the Kyoto Encyclopedia of Genes and Genomes (KEGG). This analysis resulted in the identification of PI3K/AKT signaling as a potential target. We validated the involvement of PI3K/AKT signaling in DSY against EndMT in treating AS and found that PI3K/AKT was the downstream signaling pathway of LASP1.

## Materials and methods

### Screening bioactive ingredients of Dan-Shen-Yin

The compounds in DSY were acquired using several databases including SymMap (Wu et al., 2019) (www.symmap.org), TCMSP(Ru et al., 2014) (tcmsp-e.com), TCMID (Xue et al., 2013) (http://47.100.169.139/), HERB (Fang et al., 2021) (herb.ac.cn) and ETCM (Xu H. Y. et al., 2019) (http://www.tcmip.cn/ETCM/). The SMILES structures of the compounds were obtained from PubChem (https://pubchem.ncbi.nlm.nih.gov/). The parameters of oral bioavailability (OB) ≥20% in TCMSP, quantitative estimate scores ≥0.49 in ETCM (Xu H. Y. et al., 2019), the accepted result of drug-like soft filter in FAFDrugs4 (Lagorce et al., 2017) (https://fafdrugs4.rpbs.univ-paris-diderot.fr/), and ADME with ‘high’ GI in SwissADME (Daina et al., 2017) (http://www.swissadme.ch/) were designated as thresholds to screen for potential bioactive compounds. In addition, we included the ingredients not screened by the databases, but mentioned in the literature.

### Identification of potential Dan-Shen-Yin targets in endothelial-to-mesenchymal transition and atherosclerosis

The potential targets of DSY compounds were collected by manually searching the HERB database, TCMSP database, TCMID database, SymMap database, as well as from CNKI (https://www.cnki.net/) and PubMed. Because we were unable to obtain the targets of some of the compounds from the databases or the literature, we then used SwissTargetPrediction (http://swisstargetprediction.ch/) to predict targets based on structurally similar molecules. Targets with probability ≥0.7 were retained. EndMT and AS target genes were searched from DisGeNET database (Pinero et al., 2020) (https://www.disgenet.org/), GeneCards database (Rebhan et al., 1998) (https://www.genecards.org/) and DrugBank database (Wishart et al., 2018) (https://go.drugbank.com/). The overlapping genes among targets of DSY, EndMT and AS were visualized by Jvenn (Bardou, Mariette et al., 2014) (http://jvenn.toulouse.inra.fr/app/example.html).

### Network construction and correlation analysis

The component-target network was constructed by Cytoscape (v3.9.1) and the degree value of each node, which reflects the importance of components or targets, was calculated by Network Analyzer. PPI data were acquired from Cytoscape plug-in BisoGenet (Martin et al., 2010) which includes the data from several major repositories of PPI databases such as the database of interacting proteins, biomolecular interaction network database, human protein reference database, BioGRID, and molecular interaction database.

In order to study PPIs at the system level, we then merged the PPI networks of DSY, EndMT, and AS by Cytoscape, and the Cytoscape plugin, cytoNCA (Tang et al., 2015), was used to calculate the topological properties of each node, including degree, betweenness centrality (BC), closeness centrality (CC), local average connectivity (LAC), eigenvector, and network. The nodes with topological importance in the network were screened by the topological properties mentioned above.

### Gene ontology and Kyoto encyclopedia of genes and genomes pathway enrichment analysis

GO term enrichment analysis was performed using the R package clusterProfiler. Pathway enrichment gene symbols were converted to entrez ID with the R package org.Hs.eg.db (Version3.10.0). All of the targets in the networks were also subjected to KEGG pathway enrichment analysis. The significant terms and pathways were selected according to adjusted *p* value < 0.05, and the associated GO terms and KEGG pathways were ranked by adjusted *p* value.

### Docking simulation

Autodock vina (v1.1.2) in Autodock tools (v1.5.7) (Trott and Olson 2010) was used to perform molecular docking simulations to predict the binding abilities between the bioactive ingredients in DSY and the target genes. The 3D crystal structures of target proteins were screened using the PDB database (http://www.rcsb.org/pdb/). The 3D structures of the bioactive ingredients in DSY were downloaded from PubChem database and ZINC database (https://zinc12.docking.org/) (Irwin et al., 2020). The proteins were dismissed water and added hydrogens followed by the charge were calculated before docking. For those proteins had no known active site cavity docking, a grid box was used to wrap the entire protein. Otherwise, the grid box was centered in a middle of the identified cavity. Twenty models were generated and the maximum affinity values were calculated. The higher the absolute affinity value, the stronger the binding affinity of DSY components with proteins.

### Preparation and quality control of ethyl acetate extract of Dan-Shen-Yin

*Salvia miltiorrhiza* was bought from Kangmei Pharmaceutical Co.,Ltd., (#210800301), Fructus amomi was purchased from Sinopharm Holding Shenzhen Medicinal Materials Co., Ltd.(#210502), sandalwood was obtained from Lingnan Traditional Chinese Herbal Medicine Co., Ltd. (# 2006001). In total, 200 g of *Salvia miltiorrhiza*, 30 g fructus amomi and 30 g of sandalwood (following the ratio of usage 30 g of *Salvia miltiorrhiza*, 4.5 g of fructus amomi, and 4.5 g of sandalwood in a single formula) were extracted in 500 ml of ethyl acetate for 1 h with heating and refluxing. The extract was collected into a clean beaker and filtered. Afterwards, the extract was placed in an ultra-clean tube and evaporated to dryness. The dried powder was dissolved in DMSO and stored at -20°C for later usage. The stability of the product was verified by high performance liquid chromatography (HPLC) (Figure 4).

### Cell culture

Human umbilical vein endothelial cells (HUVECs) were purchased from Cyagen (#HUVEC-2001). The HUVECs were cultured in a 1% gelatin coated plate in complete endothelial cell medium (ECM, #1001, ScienCell) containing 5% FBS, and endothelial growth factor and were maintained in a humidified chamber with 5% CO_2_ at 37°C. Human umbilical vein smooth muscle cells (HVSMCs) were obtained from Otwo Biotech (#HTX2305) and maintained in DMEM supplemented with 10% FBS and 1% P/S. To establish the EndMT model, HUVECs were stimulated with 10 ng/ml TGF-β2 (#100-35B, Proteintech) in conditioned medium supplemented with 2.5% FBS for four consecutive days. For DSY application, HUVECs pretreated with TGF-β2 for 2 days were incubated with different doses of DSY for a further 2 days.

### siRNA-mediated down-regulation of LASP1 in HUVEC

LASP1 siRNAs (#258:GAA​CUA​CAA​GGG​CUA​CGA​GAA; #193: CUG​GAU​AAG​UUC​UGG​CAU​AAA; #446:CCG​AGC​UCC​AGA​GAA​UCA​AGA) and non-targeting siRNA (siNT: UUC​UCC​GAA​CGU​GUC​ACG​U) were obtained from Sangon Biotech. A total of 25–100 nM of specific or siNT was introduced into the cells using RNATransMate (#E607402, Sangon Biotech) according to the manufacturer’s recommendations.

### Viable cell counting by CCK-8 assay

HUVECs were plated in 96-well plates in complete ECM medium and cultured overnight. The following day, cells were changed to ECM medium containing 2.5% FBS and were supplemented with 10 ng/ml TGF-β2 for 1 day. Then different doses of DSY as well as *Salvia miltiorrhiza*, sandalwood and fructus amomi were added for an additional 2 days. For IC50 analysis, cells were treated with different doses of DSY for 1 day, then 10 μl of CCK-8 assay reagent (#GK10001, GLPBIO, United States) was added to each well. The absorbance at 450 nm was measured using a spectrophotometer. Linear-regression analysis in Graphpad Prism 7 was performed to calculate IC50 value.

### Real-time quantitative PCR

HUVECs were homogenized in Trizol reagent (Thermo-Fisher Scientific) and total RNA was prepared using the EZ-press RNA purification Kit (#B0004DP, EZBioscience) according to the manufacturer’s procedure. Reverse transcription was performed using the Color Reverse transcription kit (#A0010CGQ, EZBioscience) following the manufacture’s instructions. RT-qPCR was conducted using 2xColor SYBR green qPCR mix (#A0012-R2, EZBioscience) on a C1000 thermocycler (Bio Rad), and measurements were normalized to the expression of the housekeeping gene GAPDH. The relevant primers are shown in Supplementary Table S1. The expression data are expressed as 2^−ΔΔCT^.

### Western blot analysis

Cells were rinsed with cold PBS and lysed in SDS buffer (#P0013G, Beyotime) containing 1x proteinase inhibitor cocktail and 1x phosphatase inhibitor cocktail. The determination of protein concentration was performed by Bradford protein assay. Equal amounts of protein were loaded and separated by 10% polyacrylamide gel electrophoresis, and proteins were transferred to a PVDF membrane by wet electroblotting. The membrane was incubated with anti-SM22α (1:1000, #10493-1-AP, Proteintech), anti-LASP1 (1:1000, #10515-1-AP, Proteintech), anti-AKT1 (1:1000, #60203-2-lg, Proteintech), anti-phospho-AKT (Ser473) (1:1000, #AF0016, Affinity Bioscience), and anti-β-actin (1:1000, #66009-1-Ig, Proteintech) at 4°C overnight, followed by washing and incubation with secondary anti-mouse, -goat, and -rabbit antibodies (1:1000, Abcam). Immunoreactive bands were detected.

### Immunofluorescence

HUVECs were fixed in 4% PFA for 15 min at RT and washed 3x with PBS containing 1% BSA followed by mild permeabilization with 0.1% Triton X-100 (#ST795, Beyotime, Biotechnology) for 10 min at 4°C. After three washes, cells were incubated with rabbit anti-SM22α (1:50), rabbit anti LASP1 (1:50) and goat anti-VE-cadherin (1:200, #AF1002, R&D Systems) overnight at 4°C. After four washes, samples were further incubated with appropriate AlexaFluor-coupled secondary antibodies (1:1000, #ab150129, Abcam) for 1 h at RT. Cells were then washed and mounted in mounting medium (FD8396, Hangzhou Ford Biological Technology Co., LTD).

### Statistics

Statistical analyses were carried out using GraphPad Prism 7 software (GraphPad Software, La Jolla, CA, United States). The differences among more than two groups were compared using one-way ANOVA followed by Tukey’s post-hoc test. A *p* value less than 0.05 was considered to be significant. Data are shown as the mean ± SEM from at least three independent experiments.

## Results

### Bioactive ingredients in Dan-Shen-Yin

A total of 113 compounds from *Salvia miltiorrhiza*, 97 compounds from fructus amomi and 38 compounds from sandalwood were extracted from SymMap, TCMSP, HERB, TCMID and ETCM databases. In addition, another four components from *Salvia miltiorrhiza* including caryophyllene oxide, rosmarinic acid, ailanthoidol, salvianolic acid L as well as three ingredients in fructus amomi including α-copaene, 4-methoxycinnamic acid, and 4-hydroxycinnamic acid were retrieved from the literature and included for subsequent analysis. A total of 227 ingredients were identified after merging and removing the duplicates. A detailed list of DSY ingredients is given in Supplementary Table S2.

### Targets of compounds in Dan-Shen-Yin, endothelial to mesenchymal transition, and atherosclerosis

From the bioactive ingredients in *Salvia miltiorrhiza*, fructus amomi, and sandalwood, 3478, 3968, and 353 targets were identified, respectively (Supplementary Table S3). Because some ingredients shared the same targets, a total of 4,147 targets in DSY were identified after removing duplicates. The targets of EndMT (475) were obtained from GeneCards, DrugBank database and literature by manual screening (Supplementary Table S4). The targets of AS (4,772) were identified from DisGeNET, DrugBank and GeneCards database (Supplementary Table S5). The shared targets of DSY, EndMT and AS were eventually refined to 154 (Supplementary Figure S1A and Supplementary Table S6). Thereafter, the bioactive ingredients and their predicted shared targets were used to generate the compounds—target network with Cytoscape 3.9.1 (Supplementary Figure S1). The network contained 4,379 nodes and 9,725 interaction edges. The degree value of a node corresponded to its importance in the network. The average degree value of the compounds was 6.5, and the degree value of 25 compounds was >9 (Supplementary Table S7), indicating that those compounds were the potential bioactive ingredients in DSY that targeted EndMT in the treatment of AS.

### Protein-protein interaction network construction and functional enrichment analysis

**FIGURE 1:**
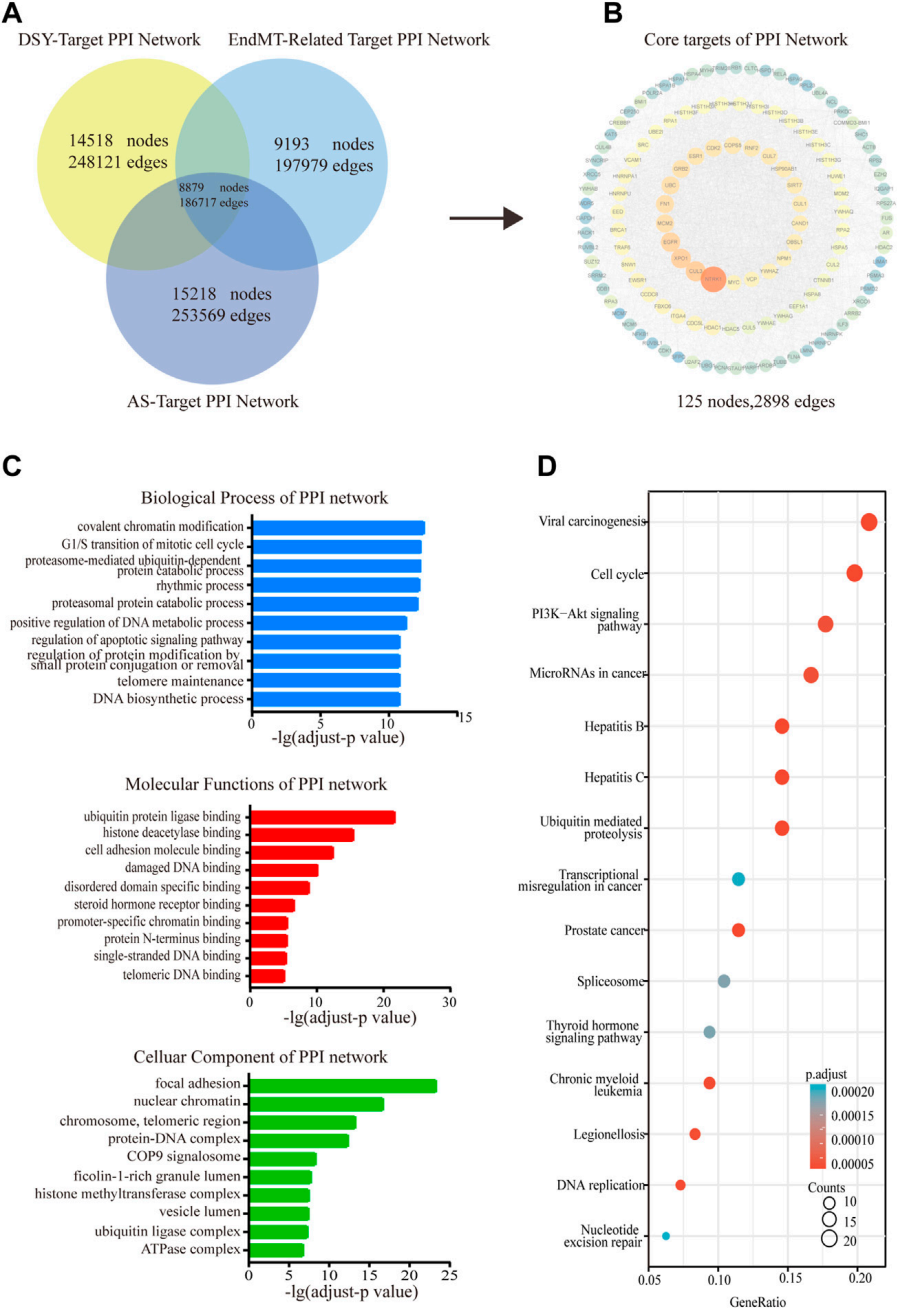
Functional enrichment analysis of the PPI network. **(A)** The shared targets of PPI with regard to DSY, EndMT and AS. **(B)** The PPI network of DSY regulating EndMT in treating AS. **(C)** GO ontology analysis of biological process, molecular functions, and cellular components. **(D)** KEGG enrichment analysis. The *X*-axis indicated the counts of the target symbols in each pathway; the *Y*-axis represented the main pathways (*p* < 0.05).

The shared targets of DSY, EndMT and AS were input into the BisoGenet plugin in Cytoscape to create a PPI network containing 8,879 nodes and 186,717 edges (Figure 1A). Next, we selected nodes that met the following conditions including degree, value, BC, CC, LAC, eigenvector, and network, and lastly we constructed a network comprising 125 nodes and 2,898 edges (Figure 1B, Supplementary Table S8). The candidate targets of the PPI network were then explored by GO and KEGG enrichment analysis. The GO category results suggested that the predicated targets were highly involved in multiple biological processes involving covalent chromatin modification, the mitotic cell cycle, catabolic processes (proteasome-mediated, ubiquitin-dependent protein catabolic, proteasomal protein catabolic process), rhythmic process, metabolic process, etc. The relevant molecular functions included chromosome binding (ubiquitin protein ligase binding, histone deacetylase binding, promoter specific chromatin binding), cell adhesion molecule binding, DNA binding (damaged DNA binding, single-stranded DNA binding, telomeric DNA binding), steroid hormone receptor binding, and protein N-terminus binding. In addition, the cellular components were markedly linked to focal adhesion, chromosome-related complex (nuclear chromatin, chromosome, telomeric region, histone methytransferase complex, ubiquitin ligase complex), protein complexes with DNA (protein-DNA complex, COP9 signalosome, ATPase complex), and lumen organization (ficolin-1-rich granule lumen, vesicle lumen) (Figure 1C). KEGG enrichment analysis suggested that the involved signaling pathways were mainly classified into cancer (viral carcinogenesis, miRNAs in cancer, prostate cancer, transcriptional misregulation in cancer, chronic myeloid leukemia), cell cycle including DNA replication, PI3K/AKT pathway, inflammation (hepatitis B, hepatitis C, legionellosis), ubiquitin mediated proteolysis, and DNA repair (Figure 1D).

### Network of Dan-Shen-Yin ingredients with PI3K pathway signals

**FIGURE 2:**
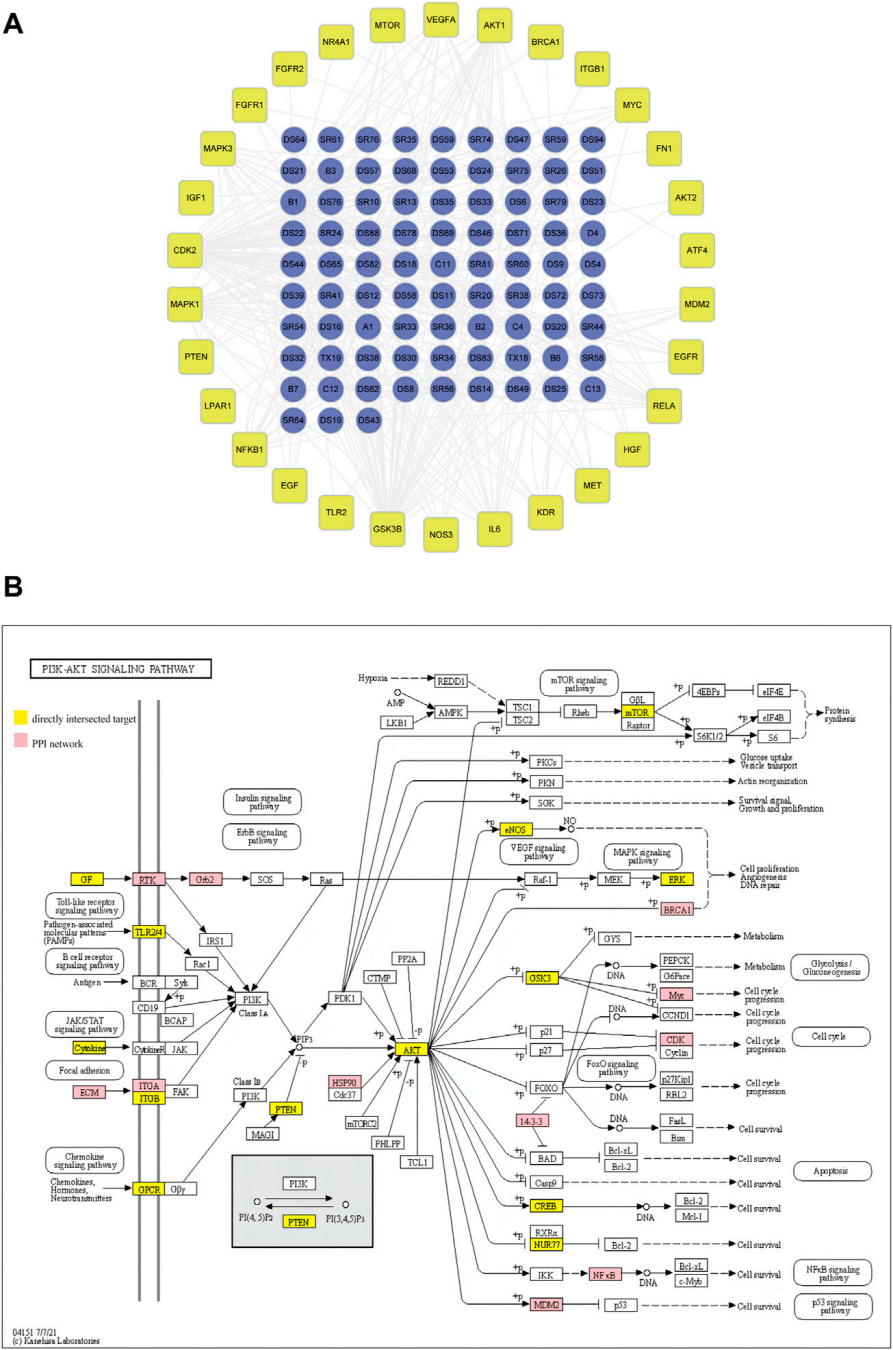
Screening of PI3K/AKT signaling pathway for regulation of EndMT in AS. **(A)** network of bioactive ingredients with PI3K/AKT targets extracted from directly intersected and PPI core targets. **(B)** The specific position and function of PI3K/AKT targets in signaling pathways. The red nodes and the yellow nodes represent the specific position of targets screened from PPI and from directly intersected targets respectively in the pathways.

As shown in Figure 1D, the PI3K/AKT pathway was enriched in the PPI network as analyzed by the KEGG. We then established a network of target genes in the PI3K/AKT pathway with the bioactive ingredients in DSY based on the obtained direct targets and PPI hub genes (Figure 2A). From this analysis, we found that GSK3β, CDK2, MAPK1, AKT1, MAPK3, IL-6, and NF-κB were the targets of over 15 bioactive ingredients, suggesting that these genes may be the primary targets. For a clearer presentation, the targets from the PPI network were colored red while the targets from the directly intersected network were colored yellow in the signaling pathway diagram in Figure 2B. It can be seen that PI3K/AKT-related targets were involved in multiple pathways, such as the toll-like receptor signaling pathway, B cell receptor signaling pathway, JAK/STAT signaling pathway, focal adhesion, and chemokine signaling pathways (Figure 2B). Since focal adhesion was one of the main cellular components by GO categorical analysis as shown in Figure 1B, we hypothesized that integrin-mediated PI3K/AKT activation was the main target of DSY for regulating EndMT in AS.

### Effect of Dan-Shen-Yin on endothelial to mesenchymal transition

**FIGURE 3:**
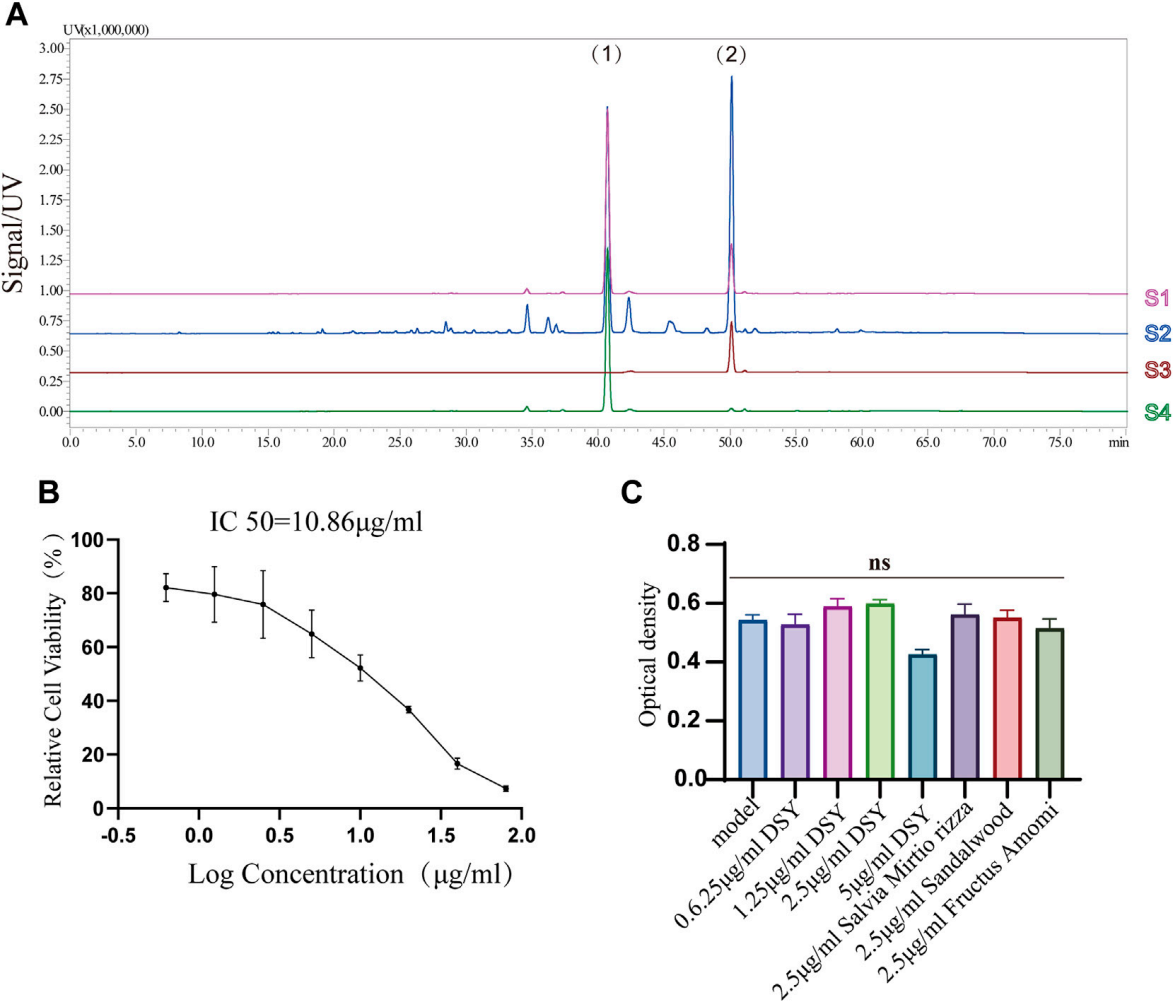
**(A)** HPLC chromatographic fingerprints of DSY. (1) and (2) represent cryptotanshinone and tanshinone IIA individually. S1: mixture of tanshinone IIA and cryptotanshinone; S2: DSY; S3: tanshinone IIA; S4: cryptotanshinone. **(B)** HUVECs were treated with different doses of DSY for 24 h followed by CCK-8 analysis. IC50 was calculated by linear-regression. **(C)** Cell viability influenced by DSY in the presence of TGF-β2 was determined by CCK-8 analysis.

**FIGURE 4:**
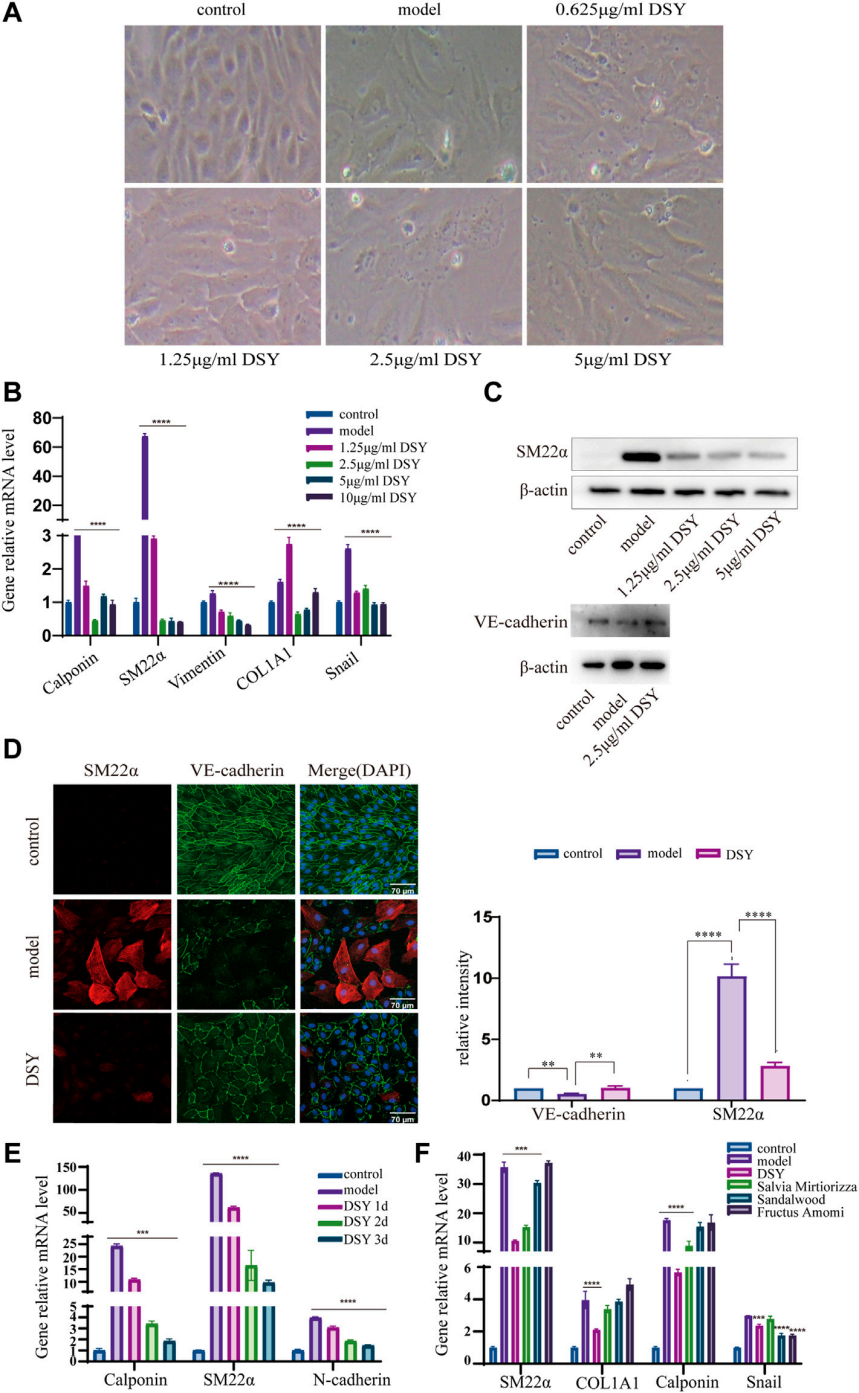
The effect of DSY on EndMT. **(A–E)** HUVECs pretreated with 10 ng/ml TGF-β2 for 2 days were incubated with different doses of DSY for another 2 d. **(A)** Representative phase contrast microscopy images. **(B)** RT-qPCR analysis of SM22α, calponin, vimentin, COL1A1, and Snail. **(C)** Western blot analysis was conducted to detect SM22α and VE-cadherin expression in DSY-treated cells. (**D, right panel**) Representative confocal images of HUVECs immune labeled with SM22α and VE-cadherin. (**D, left panel**) Relative intensities of SM22α and VE-cadherin were quantified by Fiji software. **(E)** HUVECs were pretreated with TGF-β2 for 3, 2, and 1 day followed by stimulation with 2.5 μg/ml DSY for another 1, 2, and 3 days. RT-qPCR analysis of SM22α, calponin and N-cadherin was demonstrated. **(F)** HUVECs pretreated with 10 ng/ml TGF-β2 for 2 days were treated with 2.5 μg/ml DSY, *Salvia miltiorrhiza*, fructus amomi and sandalwood for another 2 days. RT-PCR analysis of SM22α, calponin, COL1A1, and Snail was shown.

Next, we investigated the involvement of DSY in regulating the EndMT process. A total of 200 g of *Salvia miltiorrhiza*, 30 g of sandalwood, and 30 g of fructus amomi were extracted with ethyl acetate, yielding 1.4043 g of extract. The stability of the DSY extract was determined by HPLC (Figure 3A). To measure the toxicity of DSY, we conducted CCK-8 analysis and found that DSY dose-dependently reduced cell viability. The IC50 value of DSY for blocking cell proliferation in HUVECs was 10.86 μg/ml (Figure 3B). In this work, cells were incubated with TGF-β2 to induce the EndMT process and DSY had no effect on cell viability in the presence of TGF-β2 (Figure 3C). After TGF-β2 treatment, the cell phenotype underwent a significant change to a typical mesenchymal shape (Figure 4A). After DSY application, the cells adopted a cobblestone-like shape compared with TGF-β2 alone treatment (Figure 4A). Gene expression analysis by RT-PCR showed that TGF-β2 up-regulated the expression of mesenchymal markers like SM22α, calponin, collagen-type I alpha 1 chain (COL1A1), vimentin and the transcription factor, Snail, suggesting that EndMT was successfully induced. The ethyl acetate extract of DSY markedly decreased the expression of SM22α, calponin, vimentin, COL1A1, and Snail (Figure 4B). The decrease in SM22α protein expression by DSY was further confirmed by Western blot analysis (Figure 4C). From the results shown in Figures 4B,C, DSY at 2.5 μg/ml significantly decreased SM22α expression compared with DSY at 1.25 μg/ml. There was no obvious difference of SM22α expression between DSY at 2.5, 5, and 10 μg/ml; therefore, DSY at 2.5 μg/ml was used for subsequent experiments. The endothelial marker gene, VE-cadherin, was down-regulated by TGF-β2 treatment but this was reversed by DSY treatment (Figure 4C). The impact of DSY on SM22α and VE-cadherin was further confirmed by immunofluorescence staining (Figure 4D). To determine the time dependence of DSY on EndMT, HUVECs were pretreated with TGF-β2 for 3, 2, or 1 day followed by application of DSY for 1, 2, or 3 days, individually. Results indicated that DSY could inhibit EndMT in a time-dependent manner (Figure 4E). In the next experiment, the ethyl acetate extract of *Salvia miltiorrhiza*, fructus amomi and sandalwood were individually applied to HUVECs pretreated with TGF-β2. The RT-qPCR results showed that *Salvia miltiorrhiza* and sandalwood decreased SM22α, COL1A1 and calponin expression, but the effect of the single component was smaller compared to that with DSY. Fructus amomi did not affect gene expression (Figure 4F). These results suggested, on the one hand, that *Salvia miltiorrhiza* was the main component in DSY responsible for inhibiting EndMT process, while on the other hand, *Salvia miltiorrhiza,* and sandalwood showed synergistic effects against EndMT.

### Dan-Shen-Yin down-regulated PI3K/AKT pathway expression

**FIGURE 5:**
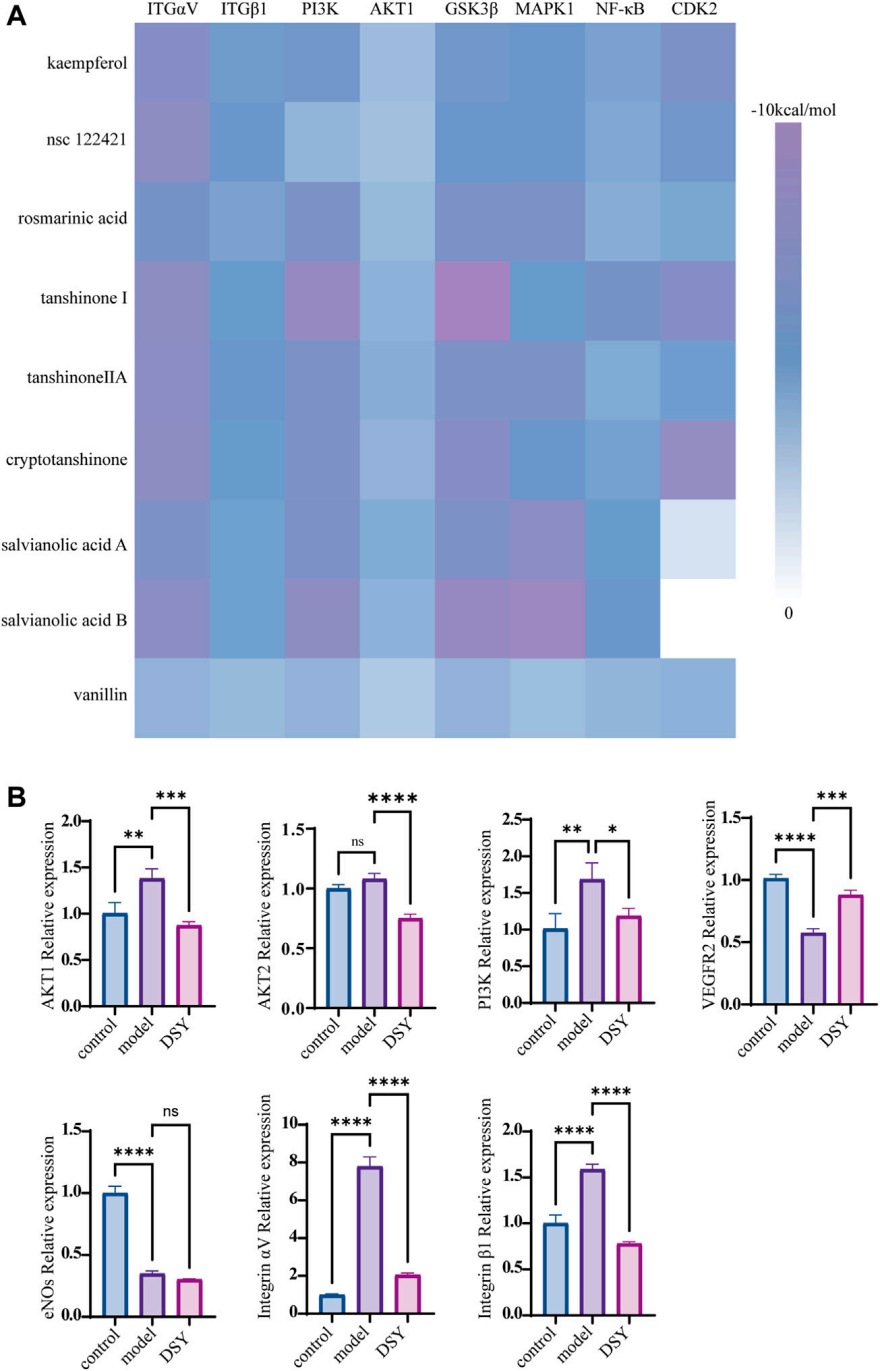
Impact of DSY on PI3K/AKT signaling. **(A)** Docking simulations of bioactive ingredients with PI3K/AKT molecules. Heat map of docking scores of binding affinity is shown. **(B)** HUVECs pretreated with 10 ng/ml TGF-β2 were subsequently stimulated with 2.5 μg/ml DSY. RT-qPCR analysis of PI3K, AKT1, AKT2, eNOS, integrin αV, and integrin β1.

As shown above, we found enrichment of the PI3K/AKT signaling pathway in the PPI network and GO enrichment analysis. We then wanted to know if DSY could directly target PI3K-AKT signaling to regulate EndMT. We chose the compounds whose degree value was greater than 9 for docking simulation to estimate their binding affinity with PI3K/AKT pathway molecules. The specific degree values of compounds are shown in Supplementary Table S8. Fructus amomi had no effect on the expression of EndMT signature genes; therefore, the compounds identified in fructus amomi were excluded (Figure 4F). As a result, kaempferol (degree = 17), tanshinone I (degree value = 17), rosmarinic acid (degree value = 16), salvianolic acid b (degree value = 15), nsc 122421 (degree value = 14), tanshinone ii a (degree value = 12), vanillin (degree value = 10), cryptotanshinone (degree value = 9), and salvianolic acid a (degree value = 9) were screened. The results indicated that the majority of bioactive ingredients had strong binding affinities with the predicted PI3K/AKT pathway genes including Integrin αV, Integrin β1, PI3K, AKT1, GSK3β, CDK2, MAPK1, and NF-κB (Figure 5A, Supplementary Figures S2, S3). In turn, we detected the expression of PI3K/AKT signals, and found that TGF-β2 could significantly up-regulate the expression of AKT1, PI3K, integrin αV, and integrin β1, while DSY was able to reverse this expression. AKT2 expression was apparently not changed by TGF-β2 treatment (Figure 5B). This result suggested that DSY could target integrin-PI3K-AKT1 signaling expression.

### Dan-Shen-Yin attenuated LIM and SH3 protein 1 expression in TGF-β2 treated cells

**FIGURE 6:**
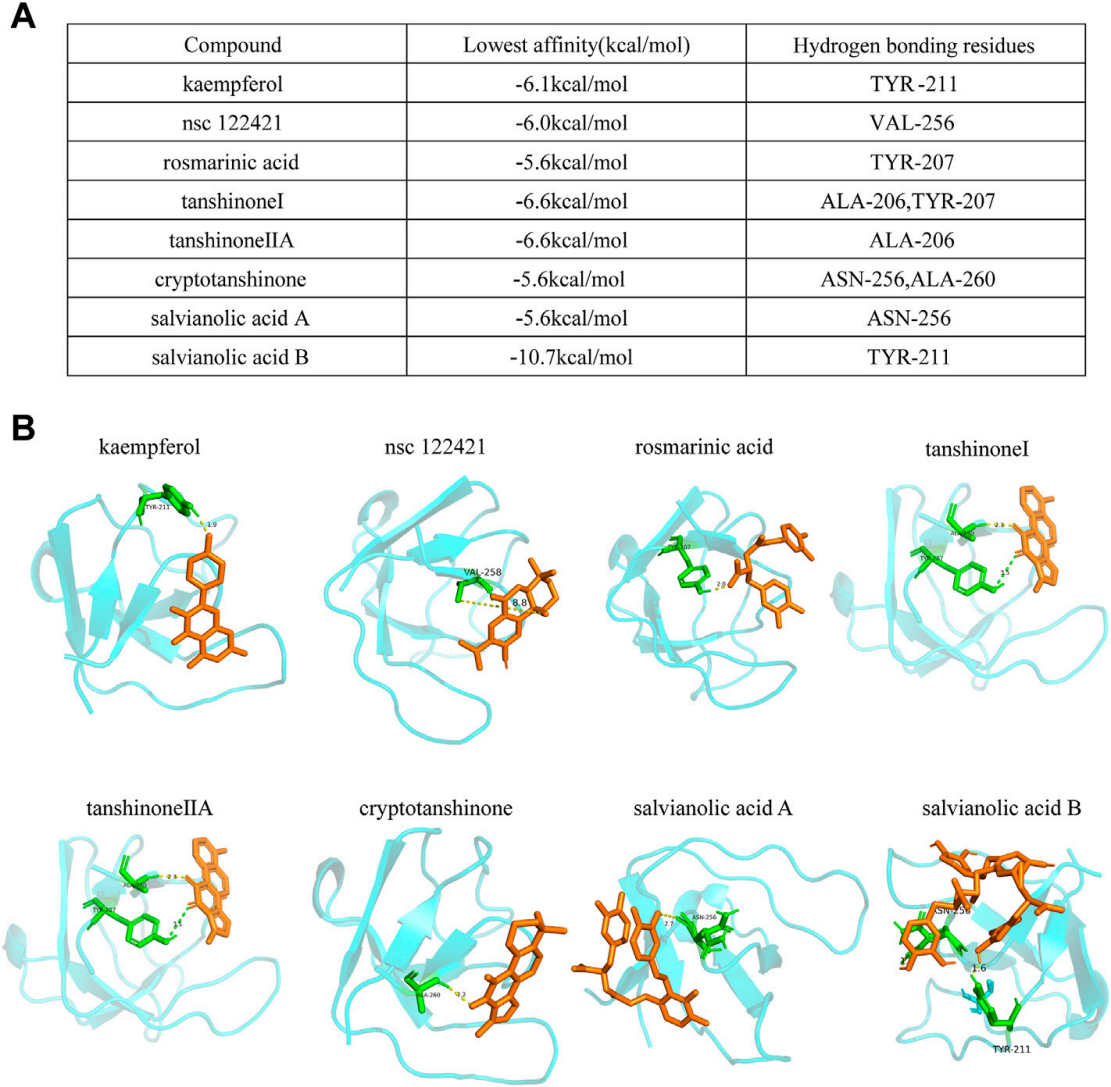
Docking simulations of bioactive ingredients with LASP1 molecule. **(A)** The binding affinities and the hydrogen bonding residues are shown. **(B)** The docking graph is shown.

**FIGURE 7:**
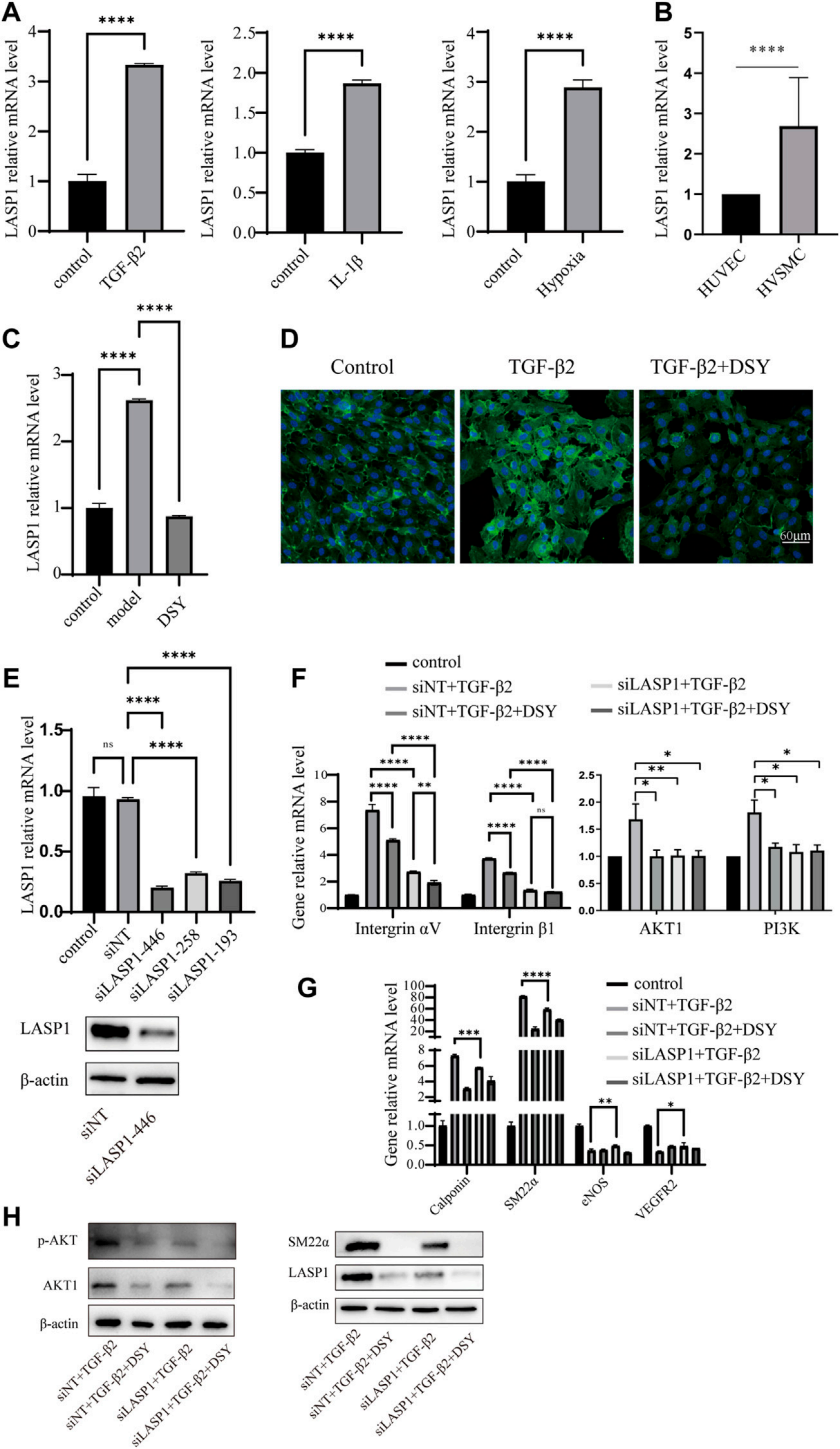
LASP1 mediated DSY down-regulation of integrin expression in TGF-β2-treated cells. **(A)** HUVECs were treated with 10 ng/ml IL-1β, TGF-β2 for 3 days or stimulated with hypoxia for 12 h. RT-qPCR analysis was then performed to detect LASP1 expression. **(B)** RT-qPCR comparison of LASP1 expression in HUVECs and HVSMCs. **(C,D)** LASP1 expression in TGF-β2-pretreated cells supplemented with different doses of DSY measured by **(C)** RT-qPCR and **(D)** immunofluorescence assay. (**E, upper panel**) RT-qPCR analysis of LASP1 expression after transfection with different siRNAs. (**E, lower panel**) LASP1 expression after transfection with siLASP-446 was checked by western blot. **(F)** HUVECs were transfected with siLASP1-446 followed by treatment with TGF-β2. One day later, 2.5 μg/ml DSY was added for another 2 d. **(F)** Analysis of integrin αV, integrin β1, PI3K, and AKT1 and **(G)** SM22α, calponin, and VEGFR2 expression by RT-qPCR analysis. **(H)** Western blot analysis of p-AKT, AKT1, SM22α and LASP1.

LIM and SH3 protein 1 (LASP1) is a specific focal adhesion protein that is induced under inflammatory conditions and in a variety of tumors (Zhang X. et al., 2017; Gao et al., 2018; Liu et al., 2018; Beckmann et al., 2021). It has been recently discovered that LASP1 can trigger the epithelial mesenchymal transition process and promote tumor progression by the PI3K/AKT pathway (Wang et al., 2014; Shao et al., 2016; Gao et al., 2018; Gao et al., 2018; Zhong et al., 2018; Zhou et al., 2018; Xue et al., 2021). Integrins serve as adhesion receptors and their functions depend on focal adhesion signaling (Quach et al., 2009). As shown above, the expression of integrin αV and integrin β1 were significantly changed by DSY in TGF-β2-treated cells; we then wanted to know if LASP1 affected integrin expression to regulate EndMT. Because *Salvia miltiorrhiza* was the component in DSY with the greatest effect on inhibiting EndMT, we first performed docking simulations to estimate the binding abilities between the bioactive ingredients of *Salvia miltiorrhiza* and LASP1. The results suggested that the majority of bioactive ingredients including kaempfero, nsc 122421, rosmarinic acid, tanshinone, tanshinone IIA, cryptotanshinone, salvianolic acid A, and salvianolic acid B exhibited strong binding abilities towards LASP1 (Figure 6). Further, we discovered that LASP1 expression was higher in HVSMCs compared to HUVECs, and its expression in HUVECs could be induced by EndMT promoting stimuli like TGF-β2, IL-1β and hypoxia (Figures 7A,B). DSY decreased LASP1 expression induced by TGF-β2, as measured by RT-PCR and immunofluorescence assay (Figures 7C,D). As expected, knock-down of LASP1 decreased the expression of integrin αV, integrin β1, PI3K and AKT1 (Figure 7F) and down-regulated the phosphorylation level of AKT (Figure 7H). But there was no obvious difference in the expression of AKT1, PI3K, and p-AKT in cells containing siNT + DSY or siLASP1+DSY, suggesting that DSY was targeting the PI3K/AKT signaling pathway through LASP1. The same effect was observed regarding the mesenchymal-specific genes, *SM22α* and *calponin*. Expression of the endothelial functional gene, *VEGFR2*, was up-regulated by LASP1 deprivation (Figures 7G,H). Taken together, these results suggested that DSY could inhibit EndMT through the LASP1/integrin/PI3K/AKT signaling pathway.

## Discussion

As the most common cardiovascular disease, AS gives rise to high morbidity and mortality worldwide. The endothelial lining on the inner surface of blood vessels has recently been determined to be transformed into mesenchymal cells through EndMT in response to various stimuli including growth factors, cytokines, hypoxia, and oscillatory flow, which contributes to the hardening and thickening of the arterial wall and plaque formation (Evrard et al., 2017; Mahmoud et al., 2017; Souilhol et al., 2018; Zhang et al., 2018). A substantial body of evidence has suggested that EndMT was the main inducer of AS development (Chen et al., 2015; Souilhol et al., 2018; Liu H. T. et al., 2021; Testai et al., 2021), and that modifying EndMT may provide a promising new therapeutic strategy. TCM has shown significant therapeutic effects against AS by the synergistic effects of multiple ingredients with multiple targets.

DSY, which is composed of *Salvia miltiorrhiza*, fructus amomi, and sandalwood, is a famous traditional Chinese formula for treating cardiovascular disease. Pharmacological studies revealed that DSY possessed protective effects against acute ischemic myocardial injury, and diabetic AS by its anti-inflammatory and antioxidant activities (Yan et al., 2011; Yan et al., 2012); but, whether DSY could treat AS by targeting EndMT was unknown. In the present study, the parameters of OB and DL in the TCMSP database were utilized to build a compound—target network comprised of 227 bioactive ingredients and 154 corresponding targets against EndMT and AS, suggesting a potential role of DSY in regulating EndMT. Experimental analysis demonstrated that DSY could decrease mesenchymal signature genes as shown by RT-qPCR, western blot and immunofluorescence assay. By creating a PPI network, GO and KEGG pathway were then used to analyze the possible signaling pathway of DSY intervention in treating AS via EndMT. The KEGG results indicated that PI3K/AKT signaling was ranked first in pathway enrichment. PI3Ks are a family of lipid kinases that phosphorylate lipids at the cell membrane followed by recruitment and activation of AKT serine/threonine kinase, resulting in activation of downstream genes (Oyagbemi et al., 2017). The PI3K/AKT pathway has been implicated in several fundamental cellular processes such as proliferation and differentiation (Nagai et al., 2013; Yu and Cui 2016), and the abnormal functioning of the PI3K/AKT pathway has been seen in the onset and progression of many diseases like AS. Many AS risk factors result in activation of the PI3K/AKT pathway such as triggering of foam cell formation, intracellular lipid accumulation, and smooth muscle cell proliferation, which all contribute to plaque formation (Brito et al., 2009; Hongo et al., 2009; Huang et al., 2013). Blocking the PI3K/AKT pathway could attenuate the inflammation process and could, in turn, decrease the AS lesion and plaque area in the ApoE^−/−^ mouse model (Liu Z. Z. et al., 2021). Therefore, modulating PI3K/AKT signaling may provide a novel strategy for treating AS. In the current study, molecular docking results indicated that the majority of the bioactive ingredients had strong binding abilities with the predicted genes involved in the PI3K/AKT pathway. The hardening and thickening of the arterial wall in AS involves local remodeling of the vessel intima, which could be induced by multiple stimuli including pro-inflammatory cytokines, LPS, oxidized LDL and mechanical stress (Chen P. Y. et al., 2019; Gorabi et al., 2021; Guijarro and Cosin-Sales 2021; Wang et al., 2021). During this process, the integrin family of adhesion receptors has been shown to regulate the endothelial phenotype and could promote inflammation and fibrotic plaque formation (Finney et al., 2017; Chen X. et al., 2019; Al-Yafeai et al., 2021). In the current work, the expression of integrin αV and integrin β1, as well as their downstream signaling proteins, PI3K and AKT1, in TGF-β2-treated cells was significantly down-regulated by DSY, suggesting that DSY could target the integrin/PI3K/AKT signaling pathway to modify EndMT.

The remaining question was how the PI3K/AKT pathway was being regulated. Many papers had reported that the PI3K/AKT pathway was activated by LASP1 in many types of cancer cells (Shao et al., 2016; Zhong et al., 2018; Zhou et al., 2018). LASP1 is a specific focal adhesion protein that is up-regulated in destructive arthritis and a variety of tumors (Zhang T. et al., 2017; Gao, Tang et al., 2018; Liu et al., 2018; Beckmann et al., 2021). In addition, LASP1 could regulate adherens junction dynamics (Beckmann et al., 2021) and has been found to manipulate the epithelial mesenchymal transition (Xue et al., 2021). The expression of integrin can be mediated by many signals such as the adherens junction cadherins (Casal and Bartolome 2018) and focal adhesion kinase (Zhao X. K. et al., 2016). These observations led us to speculate that LASP1 may regulate EndMT via the integrin/PI3K/AKT pathway. In the current study, we found that LASP1 could be highly induced by TGF-β2, IL-1β, and hypoxia, the same factors that have been reported to promote EndMT (Kumarswamy et al., 2012; Lee et al., 2012; Maleszewska et al., 2013; Zhang et al., 2018; Glaser et al., 2020). Knock-down of LASP1 decreased integrin αV, and integrin β1 expression and reduced the expression level of mesenchymal signature genes. Above all, these results suggested that LASP1 was the key target of DSY in the regulation of EndMT.

## Conclusions

TCM is a promising method for the treatment of cardiovascular disease due to its use of multiple compounds with multiple targets. We provide network pharmacology and molecular biology evidence that DSY regulates EndMT in AS mainly through the LASP1/PI3K/AKT pathway. In this work, experimental analysis uncovered that *Salvia miltiorrhiza* was the main active herb in DSY that exhibited inhibitory activity against EndMT, in agreement with the results from network pharmacology analysis that the majority of bioactive ingredients were from *Salvia miltiorrhiza*. Our work highlights the importance of *Salvia miltiorrhiza*in in treating AS, which has been suggested in other works (Li, Xu et al., 2018; Xu J. et al., 2019), but more importantly, we provided a new scenario for DSY’s targeting of EndMT for the treatment of AS. However, further extensive in vitro and in vivo experiments have to be carried out to clarify the mechanism of DSY action and, more specifically, how *Salvia miltiorrhiza* regulated EndMT. Also, the effects and mechanisms of the bioactive ingredients in *Salvia miltiorrhiza* have to be characterized. Furthermore, we found that DSY could strongly decrease mesenchymal specific genes while moderately upregulating endothelial genes, suggesting their strong ability to inhibit the transition to the mesenchymal state, while preserving a relatively refined role in recovering the endothelial function. In future, more work has to be conducted to study the anti-AS effect of DSY in combination with other compounds with pro-endothelial activity.

## Data Availability

The original contributions presented in the study are included in the article/Supplementary Material, and further inquiries can be directed to the corresponding author.

## Abbreviations

AS, atherosclerosis; BC, betweenness centrality; CC, closeness centrality; CCK-8, cell counting kit-8; DSY, Dan-Shen-Yin; EndMT, endothelial to mesenchymal transition; GO, gene ontology; KEGG, Kyoto encyclopedia of genes and genomes; LASP1, LIM and SH3 protein 1; LAC, local average connectivity; PPI, protein-protein interaction; TCM, traditional Chinese medicine.
