# Supplementary material for: Network pharmacology and experimental analysis to reveal the mechanism of Dan-Shen-Yin against endothelial to mesenchymal transition in atherosclerosis
Source: Front Pharmacol. 2022 Aug 24;13:946193. doi: 10.3389/fphar.2022.946193 (PMC9449326; doi:10.3389/fphar.2022.946193)
Supplement: Supplementary file 1 [file DataSheet1.ZIP › Supplementary Table 1.docx]

| gene | Forward primer（5'-3'） | Reverse primer（5'-3'） | |
| --- | --- | --- | --- |
| SM22α | GCAGTCCAAAATCGAGAAGAAG | | CAGAATCACGCCATTCTTCAG |
| COL1A1 | AAAGATGGACTCAACGGTCTC | | CATCGTGAGCCTTCTCTTGAG |
| Calponin | GTGAACGTGGGAGTGAAGTA | | ATGATGTTCCGCCCTTCTCTTA |
| Vimentin | TTGCCGTTGAAGCTGCTAACTACC | | AATCCTGCTCTCCTCGCCTTCC |
| VE-cadherin | AAAGAATCCATTGTGCAAGTCC | | CGTGTTATCGTGATTATCCGTG |
| LASP1 | GAACATGAAGAACTACAAGGGC | | CTTTGCCCTTGTTCTTCTCAAA |
| VEGFR2 | GGAGCTTAAGAATGCATCCTTG | | GATGCTTTCCCCAATACTTGTC |
| PI3K | GAGATTGCAAGCAGTGATAGTG | | TAATTTTGGCAGTGATTGTGGG |
| Integrin αV | ACAGGCAATAGAGATTATGCCA | | TTTATCCTGTTTCGACCTCACA |
| Integrin β1 | CTGTGATGCCTTACATTAGCAC | | ATCCAAATTTCCAGATATGCGC |
| AKT2 | AGGAGATGGAAGTGGCGGTCAG | | GCAGGATCTTCATGGCGTAGTAGC |
| AKT1 | TGACCATGAACGAGTTTGAGTA | | GAGGATCTTCATGGCGTAGTAG |
| GAPDH | GGTGAAGGTCGGAGTCAACG | | CAAAGTTGTCATGGATGHACC |

Supplementary table 1 Human gene specific primers
