# Supplementary figures and images for: Network pharmacology and experimental analysis to reveal the mechanism of Dan-Shen-Yin against endothelial to mesenchymal transition in atherosclerosis
Source: Front Pharmacol. 2022 Aug 24;13:946193. doi: 10.3389/fphar.2022.946193 (PMC9449326; doi:10.3389/fphar.2022.946193)

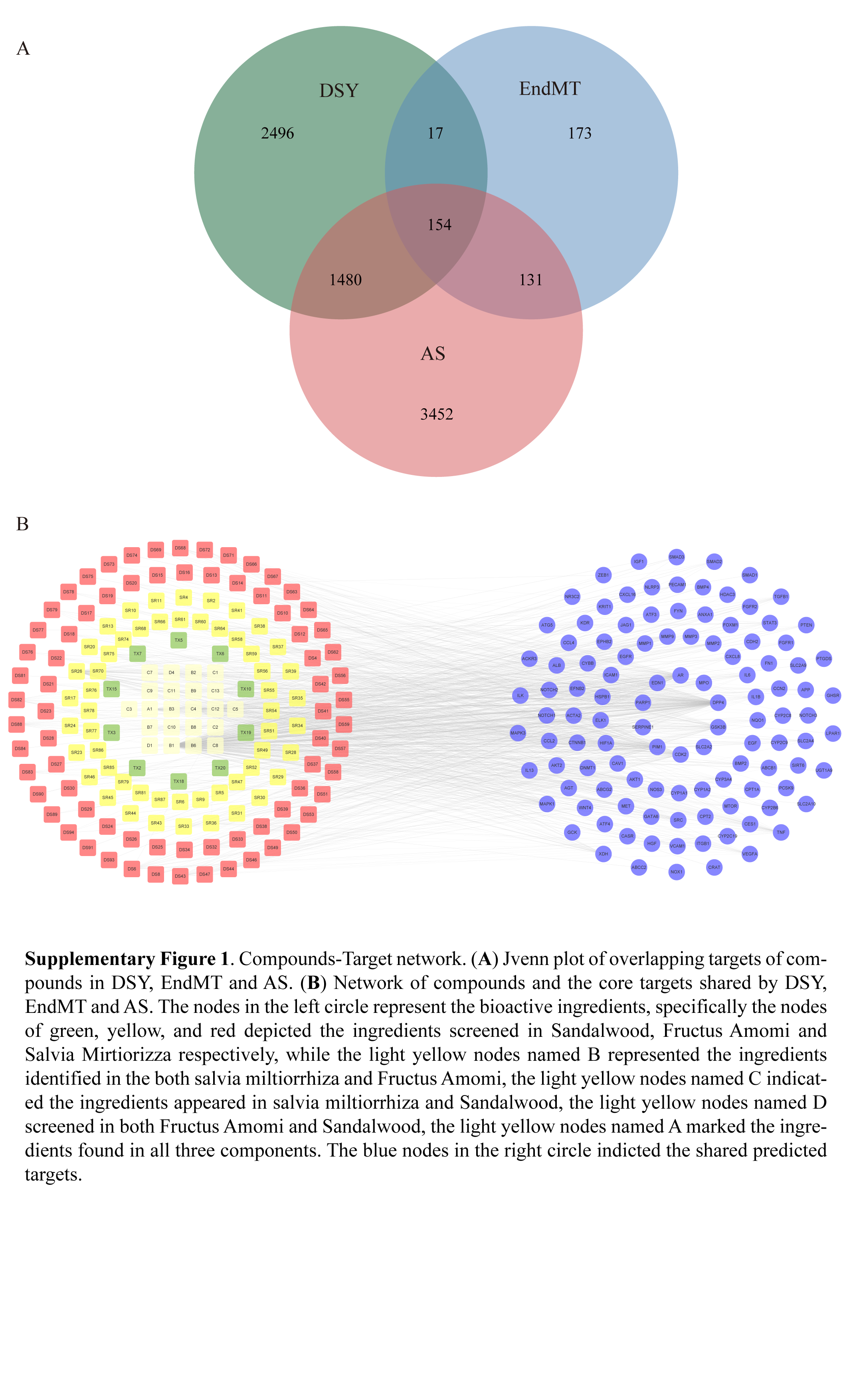

Supplement: Supplementary file 1 [file DataSheet1.ZIP › Supplementary Figure 1.tif]

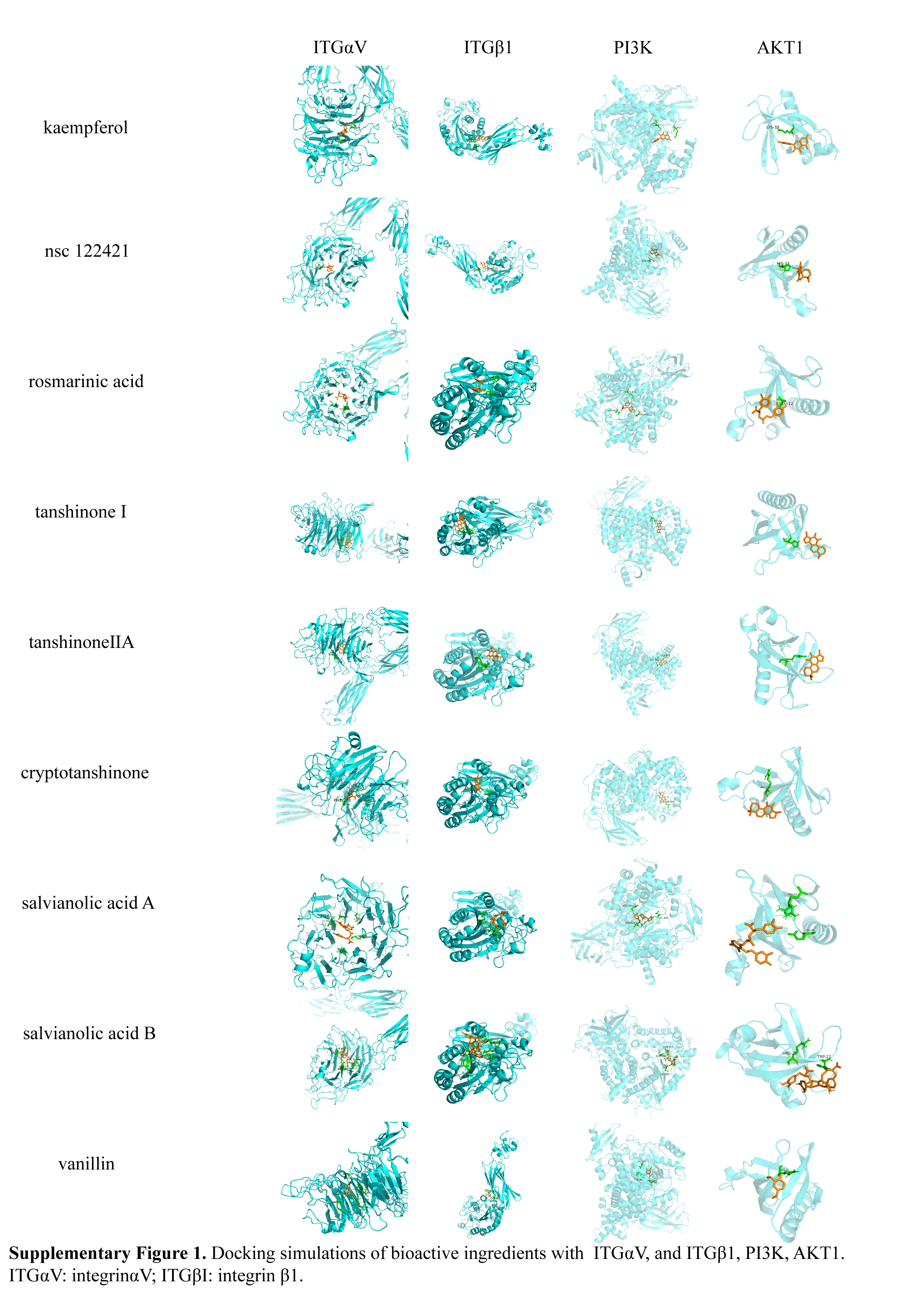

Supplement: Supplementary file 1 [file DataSheet1.ZIP › Supplementary Figure 2.tif]

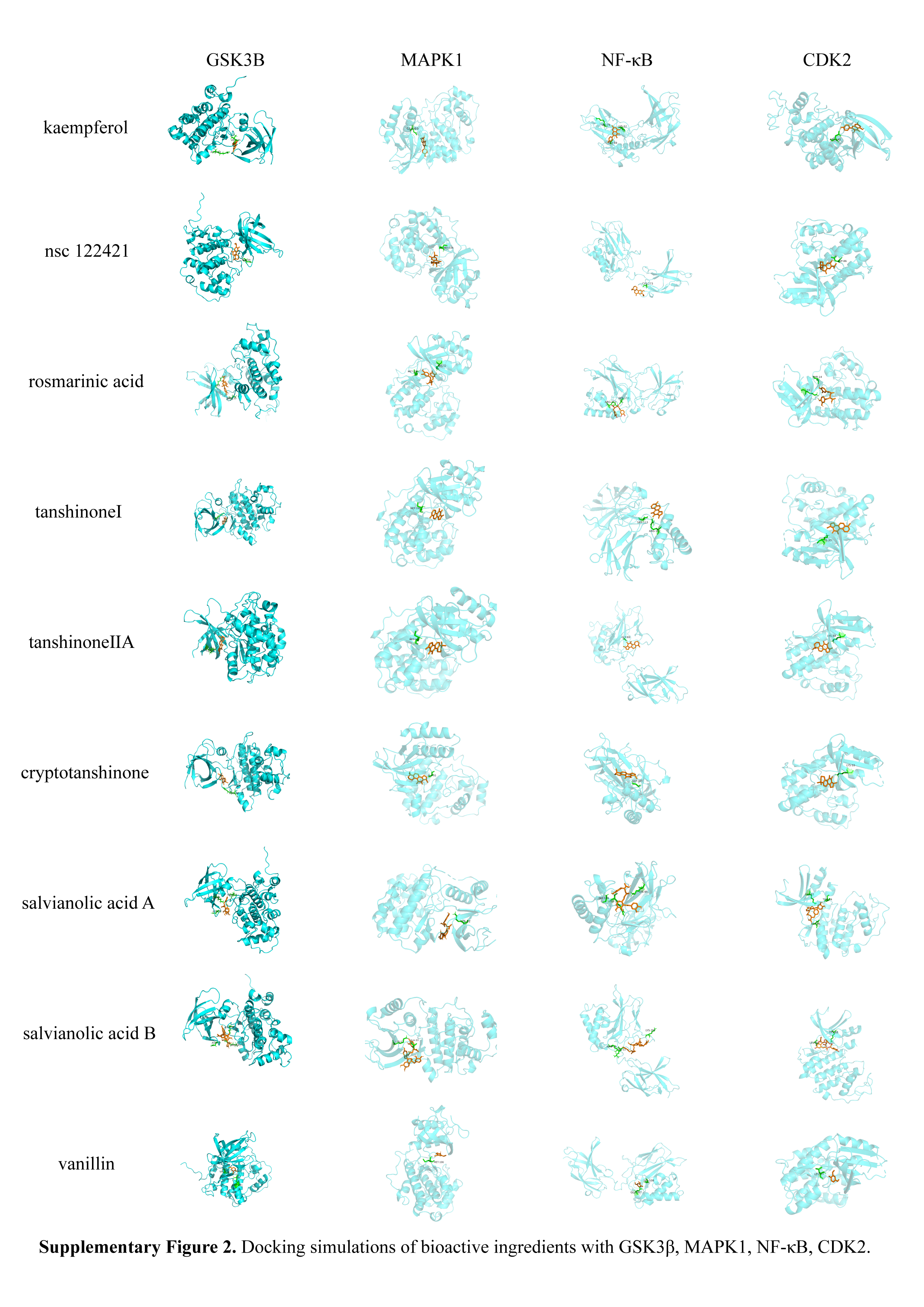

Supplement: Supplementary file 1 [file DataSheet1.ZIP › Supplementary Figure 3.tif]
